# Household Food Insecurity Is Associated with Adverse Mental Health Indicators and Lower Quality of Life among Koreans: Results from the Korea National Health and Nutrition Examination Survey 2012–2013

**DOI:** 10.3390/nu8120819

**Published:** 2016-12-16

**Authors:** Hye-Kyung Chung, Oh Yoen Kim, So Young Kwak, Yoonsu Cho, Kyong Won Lee, Min-Jeong Shin

**Affiliations:** 1 Severance Institute for Vascular and Metabolic Research, Yonsei University College of Medicine, Seoul 03722, Korea; chk@yuhs.ac; 2 Department of Food Science and Nutrition, Dong-A University, Busan 49315, Korea; oykim@dau.ac.kr; 3 Department of Public Health Sciences, BK21PLUS Program in Embodiment, Health-Society Interaction, Graduate School, Korea University, Seoul 02841, Korea; kwaksy92@daum.net (S.Y.K.); pennae@korea.ac.kr (Y.C.); chirstkw@naver.com (K.W.L.)

**Keywords:** public health, food supply, quality of life, mental health indicators, Korean

## Abstract

Food insecurity is an ongoing public health issue and contributes to mental health status. We investigated whether food insecurity is associated with inadequate nutrient intake and whether it affects mental health indicators (perceived stress/experience of depressive symptom/suicidal ideation) and quality of life (QOL) among Koreans (*n* = 5862, 20–64 years) using data from the Korea National Health and Nutritional Examination Survey (2012–2013). Household food security status was categorized as “food-secure household”, “food-insecure household without hunger”, and “food-insecure household with hunger”. Data on food insecurity, sociodemographic factors, nutrient intake, mental health indicators, and QOL were used. A logistic regression model was conducted to determine odds ratios (ORs) for psychological health. A greater proportion of food-insecure participants were nutritionally deficient compared with expectations of the 2015 Korean Dietary Reference Intakes. These deficiencies were generally higher in both “food-insecure household” groups. Both “food-insecure household” groups, particularly the “food-insecure household with hunger” group showed significantly adverse mental health status (ORs: 1.52–3.83) and lower QOL (ORs: 1.49–3.92) than did the “food-secure household” group before and after adjusting for sex, age, education, household income, smoking/alcohol consumption, physical activity, marital status, and receiving food assistance. In conclusion, food insecurity may be significantly associated with adverse mental health indicators and decreased QOL in young/middle-aged Koreans.

## 1. Introduction

Across the world, the number of hungry people is still unacceptably high, and approximately 800 million people do not eat enough food to live and an active and healthy life [1]. Therefore, food insecurity (i.e., uncertainty of having, or inability to acquire, enough food because of insufficient money or other resources) is considered an ongoing public health issue in both developed and developing countries [2,3]. The majority of hungry people live in developing countries, and 12.9% of the people in these regions remain chronically undernourished [1]. Despite remarkable economic development, the prevalence of food insecurity was as high as 14.3% among Americans in 2013 [4]. In Korea, a recent study using data from The Fifth Korea National Health and Nutrition Examination Survey (KNHANES V-3, 2012, Korea Centers for Disease Control and Prevention) reported that the prevalence of food insecurity was 11.3% among Korean adults [5].

Many studies have reported the associations between food insecurity and unfavorable health outcomes [5,6,7,8,9,10,11]. Food insecurity can cause malnutrition due to inadequate nutrient intake [5,6]. In addition, food insecurity is related to various chronic diseases, such as obesity [7], diabetes [8,9], hypertension [8], asthma [10], and cancer [11]. However, most studies have focused on socioeconomically vulnerable groups, such as children or low-income populations [6,7,10,11]. Therefore, an analysis of nationwide data is needed for a comprehensive understanding of how food insecurity has contributed to chronic diseases. 

Recently, it has been proposed that food insecurity is related to mental health problems such as mood disorders and depressive symptoms [12,13,14,15]. Moreover, food insecurity was significantly more prevalent in adults with mood disorders compared to those without mood disorders (7.3% in the general population vs. 36.1% in those with mood disorders, *p* < 0.001) [12], and a dose–response relationship between food insecurity and depressive symptoms existed (odds ratio (OR) = 3.42, 95% confidence interval (CI): 2.61–4.49) [13]. The Veterans Aging Cohort Study, which was performed on human immunodeficiency virus (HIV)-infected and uninfected veterans, conducted in 8 regions of the United States of America (Atlanta, Georgia; Baltimore, Maryland; Bronx, New York; Manhattan/Brooklyn, New York; Houston, Texas; Los Angeles, California; Pittsburgh, Pennsylvania; and Washington, District of Columbia) demonstrated that food insecurity was associated with poor medical health, with increases in reported conditions such as depression (OR = 3.00, 95% CI: 2.60–3.46) [14]. Furthermore, food insecurity was also associated with mental health status in children, adolescents and individuals with the human immunodeficiency virus [15,16,17]. A longitudinal study also suggested that food insecurity affected cognitive performance in elementary students [15]. Even though food insecurity has emerged as a contributing factor for mental health status, there is limited information regarding the relationship between food insecurity and mental health status in Korean adults. In addition to these mental health associations, several previous studies have demonstrated the relationship between food insecurity and poor quality of life (QOL) in women and ethnic minority patients with cancer [18,19]. Since it is a highly competitive society, Korea has a high rate of suicide and depression symptoms [20]. Therefore, the identification of contributing factors associated with mental health status and QOL is needed to relieve the mental health problems and improve the QOL of Koreans. Consequently, we used representative data from a nationwide survey to investigate whether food insecurity is associated with inadequate nutrient intake, and if it negatively affects the mental health indicators and QOL of young and middle-aged Koreans. 

## 2. Methods and Materials

### 2.1. Study Population

**Figure 1:**
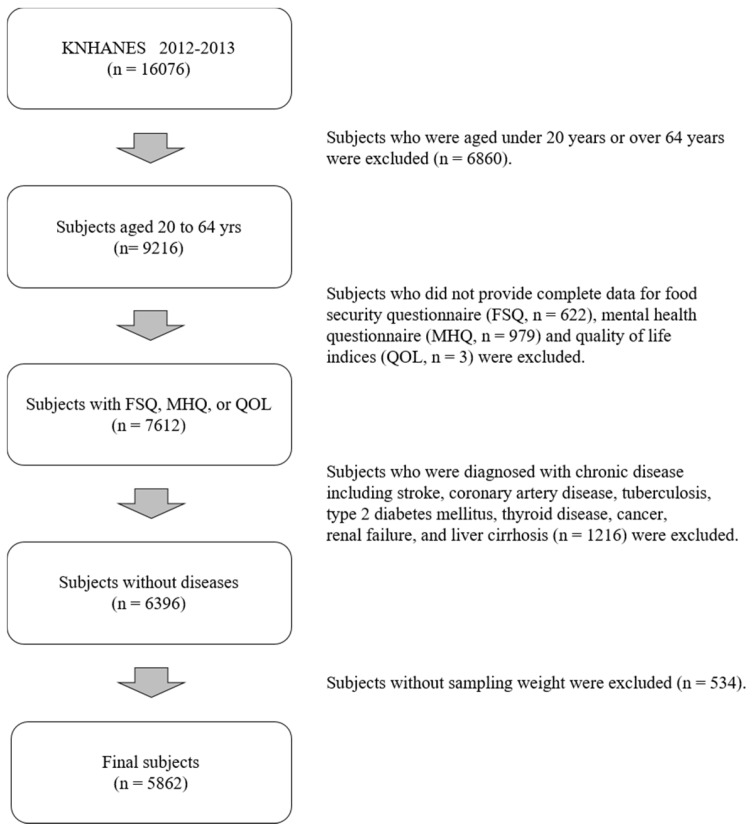
Flowchart of study population. FSQ: food security questionnaire; MHQ: metal health questionnaire; QOL: quality of life.

This study was based on data from the KNHANES (2012–2013), which is a cross-sectional and nationally representative survey. The KNHANES is conducted triannually: KNHANES I (1998), KNHANES II (2002), and KNHANES III (2005); however, the more recent surveys have been conducted annually: KNHANES IV (2007–2009), KNHANES V (2010–2012), and KNHANES VI-1 (2013). The health interview questionnaire consists of household and individual-based components collected by using self-administration or face-to-face interview methods. The household component contains information provided by an adult respondent aged ≥19 years and includes demographic variables such as income. For the sampling extraction method, 20 households from each of the 192 primary survey units were selected randomly using a stratified, multistage probability cluster sampling method that considered geographical area, age, and sex. In the selected households, household members aged over 1 year were targeted; both cohorts—8058 participants in the 2012 survey and 8018 participants in the 2013 survey—were included in this study (response rates = 80.0%, and 79.3%, respectively). The KNHANES comprises health interviews, health examinations, and a nutrition survey that were conducted by trained dietitians, medical staff, and interviewers using global standard protocols. The KNHANES collects several variables regarding participants’ demographic, social, health, and nutritional status from each component survey described above: the health interview, health examination and nutrition survey. Among the 16,076 participants, for this study we limited the analyses to adults aged 20–64 years. We excluded 1604 participants who were missing data on the following: food security questionnaire (*n* = 622), the mental health questionnaire (*n* = 979), and the QOL indices (*n* = 3). Additionally, we excluded 1216 participants who were diagnosed with chronic diseases including stroke, coronary artery disease, tuberculosis, type 2 diabetes mellitus, thyroid disease, cancer, renal failure, and liver cirrhosis to eliminate factors affecting household food security, mental health indicators, or QOL. We further excluded 534 participants whose sampling weighting information did not exist [21]. After all exclusions, 5862 participants (2278 men and 3584 women) were included for final statistical analysis (Figure 1). The KNHANES was approved by the institutional review board of the Korea Centers for Disease Control and Prevention (2012-01EXP-01-2C, 2013-07CON-03-4C). All survey participants provided informed written consent.

### 2.2. Participants’ General Characteristics

We obtained sociodemographic and anthropometric data from the KNHANES V (2012) and VI (2013). Sociodemographic data included sex, age, education level, household income, smoking status, alcohol use, physical activity, and marital status. Education level was classified into four categories: elementary school or less, middle school, high school, or university or higher. Household income was divided into quartiles for lowest, lower-middle, upper-middle, or highest. Those who had smoked more than five packs of cigarettes (100 cigarettes) during their lifetime and those who smoked daily or occasionally were defined as “current smokers” based on the answer regarding their lifetime and current smoking status. In addition, a current smoker who consumes more than 20 cigarettes per day was considered a “heavy smoker”. Regarding alcohol consumption, those who had experience consuming alcohol and a drinking frequency of more than once a month were categorized as “current alcohol users”. In addition, those who drink at least 5 glasses (man) or 2 glasses (woman) at a time, and more than twice per week were categorized as “high-risk alcohol consumers”. Those who did not have any drinking experience or drank less than once a month were categorized as “not current alcohol users”. Physical activity was assessed using self-reported data based on Korean version of condensed International Physical Activity Questionnaire whose validity and reliability was confirmed in the previous study [22]. Physical activity was divided into “exercise” or “no exercise”. Those who participated in any of these physical activities were referred to as the “exercise” group: vigorous physical activity for at least 20 min for more than 3 days per week or, moderate physical activity, or walking for at least 30 min for more than 5 days per week. On the other hand, due to the nature of the survey tool, it is not possible to distinguish between disabled and nondisabled participants. Marital status was divided into three groups: “never married” for single; “currently married (including cohabiting)”; and “formerly married” for separated, divorced, or widowed participants. Recipients of help from food assistance program were defined as those who were recently supported by the NutriPlus program, a meal service program for the elderly at a senior welfare center, a home-delivered meal service program, or a lunchbox program for children during school vacation for one year. Anthropometric measurements were conducted by trained staff members at a mobile examination center. Waist circumference (WC) was measured at midway between the rib cage and the iliac crest to the nearest 0.1 cm after breathing out normally. The measurement was performed once using controlled tapeline. Body mass index (BMI) was calculated as weight divided by the square of the height. 

### 2.3. Dietary Assessment

The diet survey was conducted by dietitians using the face-to-face interview at participants’ homes. Daily energy and nutrient intakes were obtained from the 24 h dietary recall method and estimated using the Korean Foods and Nutrients Database of the Rural Development Administration [23,24]. The absolute intakes of energy and nutrients from a 24 h dietary recall were used to estimate the proportions of energy intake deficiency (i.e., energy intake less than 75% of the estimated energy requirement) and nutrient intake deficiency (i.e., nutrient intake less than the estimated average requirement, the adequate intake, or the acceptable macronutrient distribution) by age and sex [25]. 

### 2.4. Household Food Security

Household food insecurity was surveyed by using one questionnaire in KNHANES until 2011. However, the 18-item questionnaire was recently (since 2012) added to the survey based on the U.S. Household Food Security/Hunger Survey Module to estimate food insecurity in multilateral aspects. In this study, we used data surveyed from the recently developed questionnaire including the newly added 18-item questionnaire [5,26]. The 18-item questionnaire was composed of 3 household-referenced questions, 7 adult-referenced questions, and 8 child-referenced questions. The questionnaire was completed by a major food purchaser in each household. A score of 1 was assigned to affirmative responses to food-insecure conditions and a score of 0 to all other responses in each questionnaire. The sum of the scores was used to categorize household food security status into 4 groups: food-secure household (a score of 0–2, regardless of having children), food-insecure household without hunger (a score of 3–7 with children or a score of 3–5 without children), moderate food-insecure household with hunger (a score of 8–12 with children or a score of 6–8 without children), and severe food-insecure household with hunger (a score of 13–18 with children or a score of 9–10 without children). As the number of participants in “severe food-insecure household with hunger” group was low, the “moderate food-insecure household with hunger” and “severe food-insecure household with hunger” groups were merged into a single “food-insecure household with hunger” group. Finally, our groups for household food security status were classified as follows: “food-secure household”, “food-insecure household without hunger”, and “food-insecure household with hunger”.

### 2.5. Mental Health Indicators and Quality of Life

Both mental health and QOL data were obtained from self-administered health questionnaires. The validity of the QOL questionnaire was verified in previous Korean studies [27]. For the questionnaire on mental health indicators, although there was a lack of demonstration of validity for Koreans, several previous studies used the questionnaire on mental health indicators for their analyses in Korean [28,29,30]. In the questionnaires for mental health indicators, perceived stress, experience of depressive symptoms, and suicidal ideation were used and answered as binary variables: “yes” or “no”. For perceived stress, those who reported feeling very strong or strong levels in regular life were categorized as “yes”, and feeling somewhat or a little stressed were categorized as “no”. Regarding experience of depressive symptoms, participants who reported feeling more than two weeks of continuous sadness or despair enough to disturb their usual life over the past year were regarded as “yes”. Suicidal ideation was determined based on the response to a question concerning suicidal thoughts over the past year. The EuroQoL five-dimension questionnaire (EQ-5D) developed by the EuroQoL Group was used to assess QOL. The EQ-5D comprises five dimensions: exercise ability, self-management, daily activities, pain/discomfort, and anxiety/depression. In each dimension, respondents made a choice that described their status among three categories: “no problem”, “some problem”, or “severe problem”. Based on their responses, those who had some problem or a severe problem were categorized as “problem” and those who had no problem were categorized as “no problem”.

### 2.6. Statistical Analysis

To represent the Korean population, a complex sample with applied sample weight was prepared for analysis. Statistical analysis was performed using IBM SPSS Statistics 21.0 (IBM Company, Armonk, NY, USA). Participants’ general characteristics were described as percentages and numbers for categorical variables, or as mean ± standard error for continuous variables. A chi-square test and one-way analysis of variance (ANOVA) were used to determine statistical difference in categorical and continuous variables, respectively. ORs and 95% CIs for mental health indicators and QOL were calculated using a logistic regression model. All analyses were conducted under two models: one without adjustment and one with adjustment (general linear model) for sex, age, education level, household income, smoking status, alcohol use, physical activity, marital status, and recipients of food assistance, which were proposed as confounding factors such as age, sex, BMI, marital status, education, income, alcohol behavior, smoking status, and activity levels by previous research [31,32,33,34,35,36,37,38,39] and the results from our study. However, in the analysis of ORs for daily activity, physical activity as a covariate was excluded from adjusted model due to collinearity (*p* < 0.05 was considered significant).

## 3. Results

### 3.1. Participants’ Characteristics

Participants’ basic characteristics per household food security status are shown in Table 1. In all, 7.66% were in the food insecurity group. The number of participants was the highest in the “food-secure household” group (*n* = 5413) and the lowest in the “food-insecure household with hunger” group (*n* = 68). Participants from the “food-insecure household with hunger” group were less educated, earned less household income, and currently smoked more; participants from the “food-secure household” group drank more compared with other groups (*p* < 0.05). Participants who were single or alone after marriage were prevalent in the “food-insecure household with hunger” group, and those with a married status were more prevalent in the “food-secure household” group compared to the other groups (*p* < 0.001). The proportion of receiving help from a food assistance program was higher in the “food-insecure household with hunger” group compared to the other groups (*p* < 0.001). In addition, those who had poor mental health indicators including perceived stress, depressive symptom, and suicidal ideation were more represented in the “food-insecure household with hunger” group than the other groups (*p* < 0.001). Those with low QOL (exercise ability, self-management, daily activity, pain/discomfort, and anxiety/depression) were more represented in the “food-insecure household with hunger” group than the other groups (*p* < 0.001). 

Additionally, percentages of study subjects who had deficient intakes of energy and nutrients compared with the 2015 Korean Dietary Reference Intakes (KDRIs) were generally higher in “food-insecure household” groups, particularly “food-insecure household with hunger” group than “food-secure household” group (Table 2). Precisely, energy deficiency was defined as energy intake less than 75% of the estimated energy requirement according to sex and age for Koreans. Other nutrient deficiency was defined as nutrient intake less than the acceptable macronutrient distribution, the estimated average requirement or the adequate intake according to sex and age for Koreans.

### 3.2. Odds Ratios for Mental Health Indicators per Types of Household Food Insecurity

To examine the association between household food insecurity status and mental health indicators, a logistic regression was conducted. As shown in Table 3, household food insecurity status was significantly associated with mental health before and after adjustment. Compared with the “food-secure household”, both “food-insecure household without hunger” and “food-insecure household with hunger” had poor mental health indicators; both groups showed positive associations with perceived stress before adjustment (OR = 1.56, 95% CI: 1.19–2.06, *p* = 0.001; OR = 2.15, 95% CI: 1.26–3.68, *p* = 0.005, respectively) and after adjustment (OR = 1.52, 95% CI: 1.15–2.01, *p* = 0.003; OR = 1.96, 95% CI: 1.08–3.53, *p* = 0.026, respectively). They also showed positive associations with depressive symptoms in the unadjusted model (OR = 1.68, 95% CI: 1.20–2.35, *p* = 0.003; OR = 5.77, 95% CI: 3.29–10.1, *p* < 0.001, respectively), however, after adjustment, only the “food-insecure household with hunger” group was significant (OR = 3.64, 95% CI: 2.17–6.08, *p* < 0.001). In addition, both “food-insecure household without hunger” and “food-insecure household with hunger” showed significant associations with suicidal ideation before adjustment (OR = 1.75, 95% CI: 1.13–2.72, *p* = 0.013; OR = 5.24, 95% CI: 2.77–9.94, *p* < 0.001, respectively), however, after adjustment, only “food-insecure household with hunger” showed a significant association with suicidal ideation (OR = 3.83, 95% CI: 2.02–7.23, *p* < 0.001).

### 3.3. Odds Ratios for the Quality of Life per Types of Household Food Security

The ORs for QOL per household food security status are presented in Table 4. All QOL indices were associated with household food security status. Compared with the “food-secure household” group, both “food-insecure household without hunger” and “food-insecure household with hunger” groups showed significantly higher ORs for decreased exercise ability and increased anxiety/depression before and after the adjustment. For self-management, the “food-insecure household with hunger” group had significantly higher ORs than the “food-secure household” group in the unadjusted model (OR = 8.23, 95% CI: 2.90–23.4, *p* < 0.001). In addition, the “food-insecure household without hunger” and “food-insecure household with hunger” showed significantly higher ORs for decreased daily activity than the “food-secure household” group before adjustment; however, after adjustment, only in the “food-insecure households with hunger” group was significant (OR = 3.92, 95% CI: 1.87–8.20, *p* < 0.001). Regarding pain/discomfort, both “food-insecure household without hunger” and “food-insecure household with hunger” group showed significantly higher ORs than did the “food-secure household” group; however, this was only true in the unadjusted model (*p* < 0.005).

## 4. Discussion

This study was designed to determine the effect of food insecurity on nutrient intake, mental health indicators, and quality of life. The results of this study show that food insecure participants were nutritionally deficient and showed adverse mental health status and lower quality of life. Our results are consistent with earlier findings that reported a dose–response relationship between depression and food security: the OR of depression was higher in the lowest food-secure group than the highest food-secure group (OR = 3.42, 95% CI: 2.61–4.49) [13]; and the food insecurity co-occurred with maternal depression (OR = 2.82, 95% CI: 1.62–4.93) [40] assessed by DSM-IV tool. Also, other previous studies revealed that food insecurity status change was positively associated with less depression (estimates: 0.84), assessed by composite international diagnostic interview (CIDI) [19], and demonstrated that there was strong association between food insecurity and psychological distress, assessed by the Kessler psychological distress scale (K-10) (OR = 3.4, 95% CI: 3.1–3.7) [41]. We observed that food-insecure young and middle-aged participants showed significantly higher ORs for perceived stress, depression symptoms, and suicidal ideation. A previous cross-sectional study reported an association between food insecurity and psychological distress in healthy men and women [40] and that food-insecure respondents experienced higher psychological distress compared to food-secure respondents in a clinical study on inpatients in a psychiatric hospital [42]. In line with this, low-income adults showed dose–response relationships between the level of food insecurity and the prevalence of depressive symptoms [13,19,40]. Additionally, a study using two longitudinal data sets demonstrated the relationships between food insecurity and depression in elderly adults [43]. Moreover, a cross-sectional survey on mothers of 3-year-old children found a relationship between food insecurity and anxiety disorders [40]. Another cross-sectional survey showed that food insecurity was more prevalent in adults with mood disorders than those without mood disorders [12]. Our study corroborated previous findings that food insecurity was associated with mental health indicators, even in healthy adults. Since significant associations between food insecurity and mental health indicators were maintained after adjustment for socioeconomic and lifestyle factors known to affect mental health indicators, the impact of food insecurity on mental health may be independent of these factors in this healthy population.

Food insecurity was also closely associated with lower QOL such as exercise ability and daily activity in the general population. There were relatively limited data on the relationships between food insecurity and QOL; however, an inverse association between food insecurity and QOL has been observed in women [19]. Recent studies on cancer patients also revealed that self-reported physical, functional, social, emotional well-being scores, and QOL decreased with increased levels of food insecurity [44]. The relationship between food insecurity and QOL is complicated, because QOL is composed of multilateral factors such as exercise ability, self-management, daily activities, pain/discomfort, and anxiety/depression. However, some previous studies suggested that food insecurity may affect some factors of QOL, like exercise ability and daily activity, through low-quality food intake, essential nutrient deficiencies, and poor nutritional status [45,46,47,48,49,50]. In our study, we found that the “food-insecure household” groups, particularly the “food-insecure household with hunger” group generally showed higher proportions of energy and nutrient deficiencies when compared with the 2015 KDRIs. This significantly affects mental health indicators and lowers QOL more so than it does in the “food-secure household” group.

In line with this, previous studies have documented the relationship between food intake and functional disability such as dressing, bathing, eating, and other activities [44,45,46,47]. Studies on elderly Koreans proposed that frequent consumption of dairy products, legumes, or soy products were inversely related with functional disability [45,47]. Dietary patterns are also associated with activities of daily living (ADL) and instrumental ADL (IADL) disability [47]. Evidence also exists for the harmful effects of food insecurity on nutrition status. Studies addressing Chinese elementary students and Brazil adolescents showed that food insecurity was closely related to malnutrition risk [6,48]. In our study, the proportion of participants who met the dietary reference intake for most nutrients were significantly lower in the “food-insecure household” groups, particularly the “food-insecure household with hunger” group, than the “food-secure household” group. Therefore, insufficient nutrition induced by food insecurity may be partly linked to lower QOL. However, in our study, the associations between food-insecurity, mental health indicators, and QOL were not compared between disabled and nondisabled participants, because we did not distinguish these differences among the KNHANES data. 

Our results imply that mental health indicators and QOL are associated with food insecurity and may be improved by resolving food insecurity problems. Considering that Korea has the highest suicide rate and poor mental health status among Organization for Economic Cooperation and Development countries [49,50], active intervention by the Korean government is necessary to reduce the food insecurity problem and promote better mental health. Korea has several nutrition assistance programs such as the “NutriPlus Program”, “the healthy fruit basket project”, and “free meal service”, which are provided for vulnerable populations [51,52]. Moreover, some previous studies reported the effectiveness of these programs for vulnerable Korean populations [51,53]. In our study, participation in the nutrition assistance program did not modify the association between food insecurity, mental health, and QOL. 

Our study had several limitations as well. First, this was a cross-sectional study; therefore, we cannot explain causal relationships. Second, there was a possibility of underestimating food insecurity if respondents were reluctant to answer openly to the food insecurity questionnaire. Third, the KNHANES excluded extremely food-insecure people, such as the institution-dwelling or homeless population, because it was difficult to recruit them. Fourth, the associations between food-insecurity, mental health indicators, and QOL were not compared between disabled and nondisabled participants, because we did not distinguish these differences among the KNHANES data. Finally, the questionnaire to assess mental-health problems has not been fully validated [28], even though several previous studies have used this questionnaire [29,30]. Despite these limitations, our results have important strengths when compared to previous studies’ findings. As far as we know, this is the first study demonstrating that food insecurity is a strong factor that results in adverse mental health and lower QOL in the general population of Korean adults. We analyzed a representative, large-scale data set composed of participants with homogeneous characteristics (i.e., young, middle-aged Koreans without chronic diseases). In addition, we considered various sociodemographic and health-related factors in our adjustment to minimize the possibility of confounding factors.

In conclusion, food insecurity was closely associated with insufficient nutrient intake, adverse mental health indicators, and lower QOL in young and middle-aged Korean adults. Our results provide the basic information for a health policy to prepare more effective programs to improve the mental health and QOL of individuals who have an insufficient diet. 

## Figures and Tables

**Table 1:** General characteristics of participants per types of household food security.

	Food-Secure Household (*n* = 5413)	Food-Insecure Household without Hunger (*n* = 381)	Food-Insecure Household with Hunger (*n* = 68)	*p* ^‡^
Male %, (*n*) *	39.2 (2120)	34.4 (131)	39.7 (27)	0.146
Age (year) ^†^	40.3 ± 0.2	40.1 ± 0.7	40.9 ± 1.8	0.842
waist circumference (cm)	79.9 ± 0.2	80.3 ± 0.7	80.6 ± 1.3	0.751
Body mass index (kg/m^2^)	23.7 ± 0.1	24.0 ± 0.3	23.6 ± 0.5	0.510
Education %, (*n*) ^1^				
≤Elementary school	7.4 (550)	15.0 (57)	32.4 (22)	<0.001
≤Middle school	7.0 (435)	10.8 (41)	14.7 (10)	
≤High school	44.2 (2168)	49.1 (187)	30.9 (21)	
≥University	41.5 (2256)	25.2 (96)	22.1 (15)	
Income %, (*n*) ^1^				
Lowest	6.7 (362)	19.8 (85)	48.9 (33)	<0.001
Lower middle	24.6 (1295)	44.8 (165)	34.5 (21)	
Upper middle	32.5 (1717)	25.8 (98)	13.3 (11)	
Highest	36.3 (2004)	9.7 (32)	3.4 (3)	
Current smokers %, (*n*)	25.4 (1375)	30.4 (116)	36.8 (25)	0.035
Current drinkers %, (*n*)	62.3 (3372)	53.3 (203)	58.81 (40)	0.010
Physical activity %, (*n*)	47.9 (2593)	47.0 (179)	44.1 (30)	0.851
Marital status %, (*n*) ^1^				
Single	27.5 (1020)	27.7 (82)	31.7 (16)	<0.001
Married	68.2 (4105)	56.9 (234)	38.6 (30)	
Married (alone)	4.3 (278)	15.4 (65)	29.7 (22)	
Food assistance %, (*n*)	1.0 (54)	4.7 (18)	8.8 (6)	<0.001
Mental health				
Perceived stress %, (*n*)	24.4 (1321)	33.6 (128)	41.2 (28)	<0.001
Depressive symptom %, (*n*)	9.4 (09)	15.0 (57)	38.2 (26)	<0.001
Suicidal ideation %, (*n*)	6.9 (373)	11.5 (44)	27.9 (19)	<0.001
Quality of life				
Exercise ability %, (*n*)	5.3 (287)	13.4 (51)	25.0 (17)	<0.001
Self-management %, (*n*)	1.1 (60)	1.8 (7)	8.8 (6)	<0.001
Daily activity %, (*n*)	3.1 (168)	5.8 (22)	22.1 (15)	<0.001
Pain/discomfort %, (*n*)	16.2 (877)	22.8 (87)	33.8 (23)	<0.001
Anxiety/depression %, (*n*)	8.3 (449)	15.2 (58)	30.9 (21)	<0.001

* The values of male, education, income, current smokers, current drinkers, physical activity, marital status, recipients of food assistance, mental health indicators (perceived stress, depressive symptom, and suicidal ideation), and quality of life variables (exercise ability, self-management, daily activity, pain/discomfort, and anxiety/depression) are represented as the percentage of total participants. ^†^ The values of age, waist circumference, and body mass index are represented as mean ± standard error. ^‡^ Statistical differences were determined using general linear model, complex samples crosstabs statistics for categorical variables (*p*-value < 0.05). ^1^ Total number in some variables can be different from the number of total subjects due to missing values.

**Table 2:** Proportion of participants who were energy and nutrient deficient when compared with the 2015 Korean Dietary Reference Intakes (KDRIs) per household food security status.

Variables	Food-Secure Household (*n* = 5413)	Food-Insecure Household without Hunger (*n* = 381)	Food-Insecure Household with Hunger (*n* = 68)	*p*-Value
Energy ^1^	28.1 ± 0.8	31.7 ± 3.1	43.7 ± 6.1	0.029
Protein ^2^	9.60 ± 0.5	8.70 ± 1.8	8.40 ± 4.5	0.868
Protein ^3^	18.3 ± 0.7	23.1 ± 2.7	34.7 ± 6.6	0.003
Fiber ^3^	97.6 ± 0.3	98.3 ± 0.7	98.9 ± 1.1	0.567
Calcium ^3^	66.1 ± 0.8	70.7 ± 2.9	86.4 ± 3.4	0.002
Phosphorous ^3^	7.80 ± 0.4	13.1 ± 2.1	24.5 ± 6.6	<0.001
Iron ^3^	19.6 ± 0.7	24.9 ± 2.7	29.9 ± 6.1	0.018
Sodium ^4^	6.5 ± 0.4	8.60 ± 1.8	13.0 ± 4.2	0.072
Potassium ^4^	66.0 ± 0.8	67.9 ± 2.9	84.7 ± 4.6	0.017
Vitamin A ^3^	39.6 ± 0.8	46.7 ± 3.2	55.9 ± 7.1	0.006
Vitamin B1 ^3^	17.1 ± 0.7	25.4 ± 3.2	23.8 ± 5.5	0.003
Vitamin B2 ^3^	41.3 ± 0.8	45.7 ± 3.3	60.2 ± 7.3	0.023
Niacin ^3^	24.5 ± 0.7	37.2 ± 3.4	46.1 ± 7.7	<0.001
Vitamin C ^3^	47.5 ± 1.0	59.8 ± 2.9	68.6 ± 6.4	<0.001

All values are presented as the percentage ± standard error; Statistical differences were determined using complex samples crosstabs statistics (*p* value < 0.05); ^1^ energy deficiency was defined as energy intake less than 75% of the estimated energy requirement per sex and age for Koreans; ^2^ nutrient deficiency was defined as nutrient intake less than the acceptable macronutrient distribution per sex and age for Koreans; ^3^ nutrient deficiency was defined as nutrient intake less than the estimated average requirement per sex and age for Koreans; ^4^ nutrient deficiency was defined as nutrient intake less than the adequate intake per sex and age for Koreans.

**Table 3:** Odds ratios and 95% confidence intervals for the mental health indicators per types of household food security.

	Food-Secure Household (*n* = 5413)	Food-Insecure Household without Hunger (*n* = 381)	*p*-Value	Food-Insecure Household with Hunger (*n* = 68)	*p*-Value
Perceived stress *					
Unadjusted ^†^	1.00 (ref)	1.56 (1.19–2.06)	0.001	2.15 (1.26–3.68)	0.005
Multivariable adjusted ^1^	1.00 (ref)	1.52 (1.15–2.01)	0.003	1.96 (1.08–3.53)	0.026
Depressive symptom					
Unadjusted ^†^	1.00 (ref)	1.68 (1.20–2.36)	0.003	5.77 (3.29–10.1)	<0.001
Multivariable adjusted ^1^	1.00 (ref)	1.26 (0.89–1.78)	0.185	3.64 (2.17–6.08)	<0.001
Suicidal ideation					
Unadjusted ^†^	1.00 (ref)	1.75 (1.13–2.72)	0.013	5.24 (2.77–9.94)	<0.001
Multivariable adjusted ^1^	1.00 (ref)	1.33 (0.83–2.14)	0.234	3.83 (2.02–7.23)	<0.001

* Values are presented as odds ratios (95% confidence intervals); ^†^ differences were tested using unadjusted complex samples logistic regression analysis; ^1^ differences were tested using multivariable-adjusted complex sample logistic regression analysis after adjusting for sex, age, income, education, alcohol use, smoking status, physical activity, marital status, and recipients of food assistance.

**Table 4:** Odds ratios and 95% confidence intervals for the quality of life per types of household food security.

	Food-Secure Household (*n* = 5413)	Food-Insecure Household without Hunger (*n* = 381)	*p*-Value	Food-Insecure Household with Hunger (*n* = 68)	*p*-Value
Decreased Exercise ability *					
Unadjusted ^†^	1.00 (ref)	2.78 (1.86–4.14)	<0.001	5.93 (3.15–11.2)	<0.001
Multivariate adjusted ^1^	1.00 (ref)	2.22 (1.45–3.40)	<0.001	3.22 (1.78–5.82)	<0.001
Self-management					
Unadjusted ^†^	1.00 (ref)	1.68 (0.77–3.66)	0.191	8.23 (2.90–23.4)	<0.001
Multivariate adjusted ^1^	1.00 (ref)	1.15 (0.49–2.70)	0.756	3.19 (0.94–10.8)	0.062
Decreased Daily activity					
Unadjusted ^†^	1.00 (ref)	1.90 (1.10–3.28)	0.021	9.05 (4.81–17.1)	<0.001
Multivariate adjusted ^2^	1.00 (ref)	1.21 (0.67–2.18)	0.523	3.92 (1.87–8.20)	<0.001
Pain/discomfort					
Unadjusted ^†^	1.00 (ref)	1.54 (1.13–2.08)	0.006	2.55 (1.40–4.65)	0.002
Multivariate adjusted ^1^	1.00 (ref)	1.29 (0.95–1.76)	0.108	1.69 (0.87–3.27)	0.124
Anxiety/depression					
Unadjusted ^†^	1.00 (ref)	1.97 (1.36–2.87)	<0.001	4.84 (2.74–8.54)	<0.001
Multivariate adjusted ^1^	1.00 (ref)	1.49 (1.00–2.23)	0.049	2.63 (1.52–4.55)	0.001

* Values are presented as odds ratios (95% confidence intervals); ^†^ differences were tested using unadjusted complex samples logistic regression analysis; ^1^ differences were tested using multivariate-adjusted complex sample logistic regression analysis after adjusting for sex, age, income, education, alcohol use, smoking status, physical activity, marital status, and recipients of food assistance; ^2^ differences were tested using multivariate-adjusted complex sample logistic regression analysis after adjusting for sex, age, income, education, alcohol use, smoking status, marital status, and recipients of food assistance.
